# Experimental evidence for hydrogen incorporation into Earth’s core

**DOI:** 10.1038/s41467-021-22035-0

**Published:** 2021-05-11

**Authors:** Shoh Tagawa, Naoya Sakamoto, Kei Hirose, Shunpei Yokoo, John Hernlund, Yasuo Ohishi, Hisayoshi Yurimoto

**Affiliations:** 1 Department of Earth and Planetary Science, The University of Tokyo, Bunkyo, Tokyo 113-0033 Japan; 2 Creative Research Institution (CRIS), Hokkaido University, Sapporo, Hokkaido 001-0021 Japan; 3 Earth-Life Science Institute, Tokyo Institute of Technology, Meguro, Tokyo 152-8550 Japan; 4 Japan Synchrotron Radiation Research Institute, Sayo, Hyogo 679-5198 Japan; 5 Department of Natural History Sciences, Hokkaido University, Sapporo, Hokkaido 060-0810 Japan

**Keywords:** Geochemistry, Geophysics

## Abstract

Hydrogen is one of the possible alloying elements in the Earth’s core, but its siderophile (iron-loving) nature is debated. Here we experimentally examined the partitioning of hydrogen between molten iron and silicate melt at 30–60 gigapascals and 3100–4600 kelvin. We find that hydrogen has a metal/silicate partition coefficient *D*_H_ ≥ 29 and is therefore strongly siderophile at conditions of core formation. Unless water was delivered only in the final stage of accretion, core formation scenarios suggest that 0.3–0.6 wt% H was incorporated into the core, leaving a relatively small residual H_2_O concentration in silicates. This amount of H explains 30–60% of the density deficit and sound velocity excess of the outer core relative to pure iron. Our results also suggest that hydrogen may be an important constituent in the metallic cores of any terrestrial planet or moon having a mass in excess of ~10% of the Earth.

## Abstract (web summary)

Based on diamond-anvil cell experiments and cutting-edge secondary ion mass spectrometry analyses, the authors here show that hydrogen may be an important constituent in the Earth’s core and also in the metallic cores of any terrestrial planet or moon having a mass in excess of 10% of the Earth.

## Introduction

Existing Earth’s core formation models constrained by partitioning of moderately siderophile elements suggest that metals equilibrated with molten silicates in a deep “magma ocean” under high-pressure and -temperature (*P–T*) conditions up to about 60 GPa and 4000 K^1–6^ (Supplementary Fig. 1). While hydrogen is one of the plausible impurity elements in the iron-rich core^7^, its metal–silicate partitioning under such conditions has been controversial in both experiments^8–12^ and theory^13–15^. Previous experimental studies on the metal–silicate partitioning of hydrogen were performed below 20 GPa using molten^8,11,12^ and solid^9,10^ silicates. While hydrogen has been found to be strongly siderophile under high pressure^8–10^, these results were challenged by more recent experiments^11,12^. These later studies^11,12^ found low hydrogen concentrations in iron, which may be attributed to the fact that they conducted carbon-saturated experiments, leading to carbon enrichment in metal that may hinder hydrogen incorporation. It is also possible that hydrogen escaped from their samples prior to measurements performed after they were decompressed and recovered at ambient conditions, where FeH_*X*_ is known to decompose^10^ into bcc Fe and molecular H_2_. The equilibrium hydrogen solubility in bcc Fe at an H_2_ pressure of 1 bar is limited to^16^ *X*  < 10^−5^. Loss of hydrogen from iron samples during decompression has been demonstrated in previous neutron diffraction^10^ as well as our new X-ray diffraction (XRD) measurements (Supplementary Fig. 2).

Here, we report experiments to constrain the partitioning of hydrogen between molten iron and silicate melt at high *P–T* to 60 GPa. The results demonstrate the strongly siderophile nature of hydrogen under conditions where metals segregated from silicates during Earth’s core formation, suggesting that hydrogen is an important light element in the core. In addition, hydrogen may be a major impurity element in the metallic cores of other terrestrial planets and moons whose masses are more than 10% of that of the Earth.

## Results and discussion

### Metal–silicate partitioning of hydrogen

**Fig. 1:**
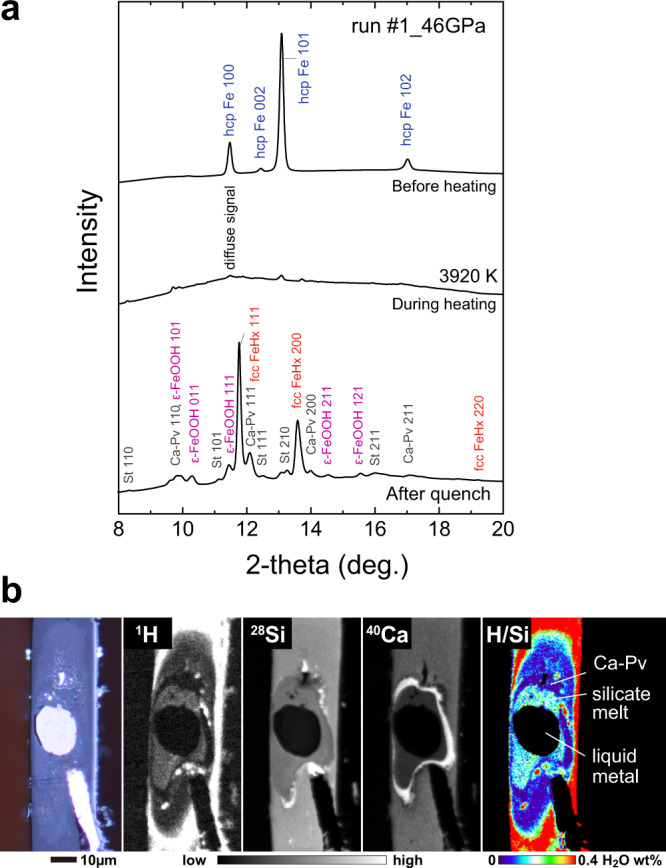
Analyses of hydrogen in metal and silicate. **a** XRD patterns collected before/during/after heating to 3920 K at 46 GPa in run #1. Both iron and silicate were molten at the center of a laser-heated spot during heating. Hydrogen concentration in liquid metal was obtained from those in fcc FeH_*X*_ and ε-FeOOH. **b** Photomicrograph (left), secondary ion images for ^1^H^+^, ^28^Si^+^, and ^40^Ca^+^ (middle), and a distribution map of water (right). Note the absence of hydrogen in metal because it escaped from iron upon releasing pressure. Hydrogen (water) content in quenched silicate melt was obtained with ±2% to ±7% relative uncertainty.

**Fig. 2:**
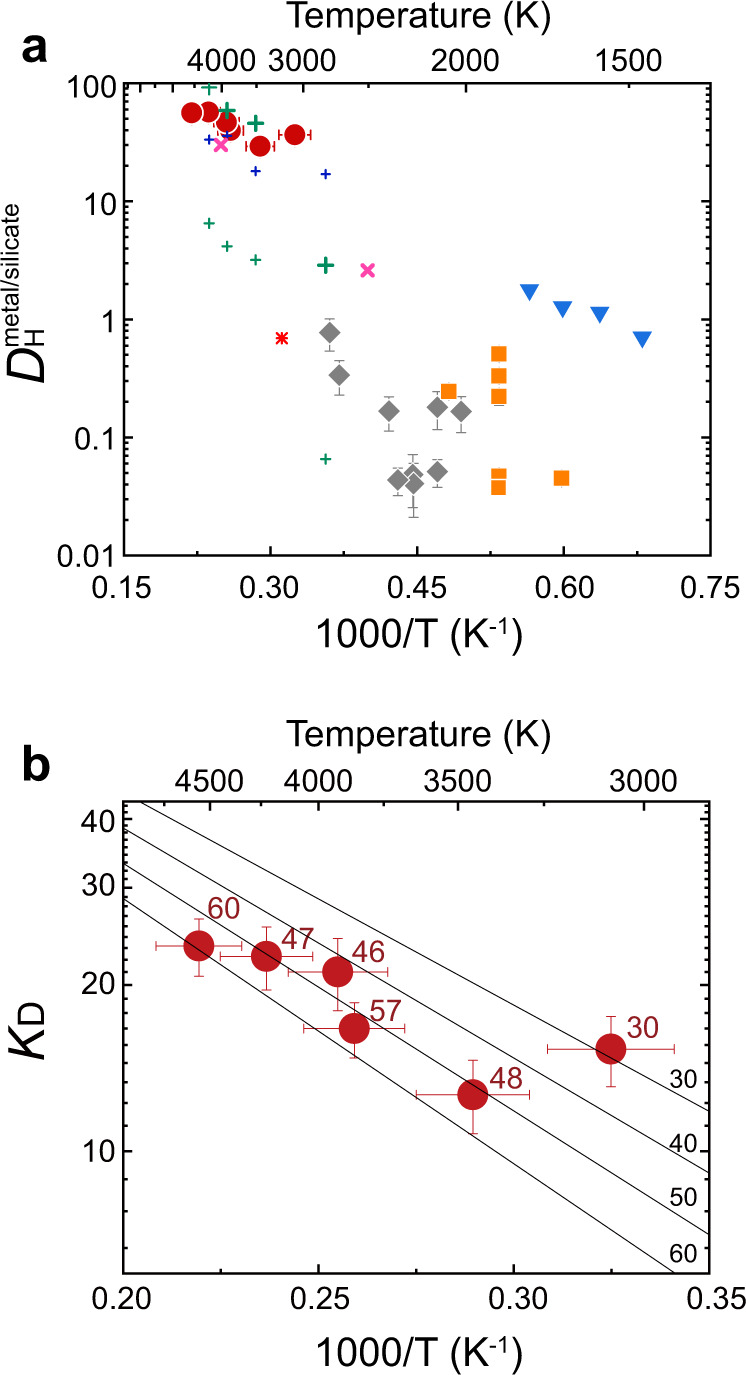
Metal–silicate partitioning of hydrogen. **a** Partition coefficient *D* (by weight) for hydrogen as a function of reciprocal temperature. The present experiments (red circles) show that hydrogen is strongly siderophile under conditions of Earth’s core formation. Recent experimental studies (gray diamonds^11^, orange squares^12^) reported *D* values lower by one to three orders of magnitude than the present results, which is likely attributed to hydrogen loss from metals during decompression. Pioneer experimental results^8^ (blue inverted triangles) and recent theoretical values (star^13^, pink crosses^15^, and blue pluses^14^ for H and green pluses^14^ for H_2_O; large and small symbols indicate maximum and minimum values, respectively) are also plotted. **b** Exchange coefficient *K*_D_ for Eq. 2. The numbers given to each datum point and regression line indicate pressure conditions.

We carried out metal–silicate partitioning experiments at 30–60 GPa and 3100–4600 K in a diamond-anvil cell (DAC) (Supplementary Table 1 and Supplementary Fig. 3) and found 5300 to 26,000 ppm H (by weight) in metal at high pressure (before decompression), on the basis of the phase proportion and lattice volume of FeH_*X*_ and ε-FeOOH that formed from liquid iron upon temperature quenching under sustained confining pressure (Fig. 1a) (see “Methods”). Note that liquid iron was likely to have quenched fully to crystals and subsequent thermal annealing in a separate experiment had a negligible effect upon hydrogen concentrations (only 6% difference)^17^. On the other hand, we obtained 90–470 ppm H (present as H_2_O) in quenched silicate melts by measuring recovered samples using a high-resolution imaging technique coupled with secondary ion mass spectrometry (SIMS) (Fig. 1b), which enabled quantitative compositional analysis of a small DAC sample (see “Methods”). In $D_{\mathrm{H}}^{{\mathrm{metal/silicate}}}$ we found ranges from 29 to 57 by weight (Fig. 2a), indicating that hydrogen is siderophile at these *P–T* conditions.

The metal–silicate partitioning of hydrogen can be expressed as a chemical reaction,

1 $${\mathrm{HO}}_{0.5}^{{\mathrm{silicate}}\,{\mathrm{melt}}} + \frac{1}{2}{\mathrm{Fe}}^{{\mathrm{metal}}} = {\mathrm{H}}^{{\mathrm{metal}}} + \frac{1}{2}{\mathrm{FeO}}^{{\mathrm{silicate{\kern 1pt}} melt}}$$

The exchange coefficient *K*_D_ is parameterized as a function of *P* (GPa) and *T*(K) with regression constants *a*, *b*, and *c* (Fig. 2b),

2 $$\begin{array}{*{20}{l}}\log _{10}K_{\mathrm{D}}\hfill & \!\!\! =\ \hfill & {\mathrm{log}}_{10}\frac{{x_{\mathrm{H}}^{\prime{\mathrm{metal}}}}}{{x_{{\mathrm{HO}}_{0.5}}^{{\mathrm{silicate}}}}} \cdot \sqrt {\frac{{x_{{\mathrm{FeO}}}^{{\mathrm{silicate}}}}}{{x_{{\mathrm{Fe}}}^{\prime{\mathrm{metal}}}}}}\hfill\\ \hfill& \!\!\!=\!\!\! \hfill & {\mathrm{log}}_{10}\frac{{x_{\mathrm{H}}^{\prime{\mathrm{metal}}}}}{{x_{{\mathrm{HO}}_{0.5}}^{{\mathrm{silicate}}}}} + \frac{1}{4}\Delta {\mathrm{IW}}\\ & \!\!\!=\!\!\! \hfill & a + \frac{b}{T} + c \cdot \frac{P}{T}\hfill\end{array}$$

where *x* represents molar fraction (see “Methods” for *x*′) and the effects of Si, O, and C are not parameterized explicitly. Oxygen fugacity *f*O_2_ relative to the iron-wüstite (IW) buffer is approximated as $\Delta {\mathrm{IW}} \approx 2\log _{10}\left( {x_{{\mathrm{FeO}}}^{{\mathrm{silicate}}}/x _{{\mathrm{Fe}}}^{\prime{\mathrm{metal}}}} \right)$ following previous studies^2,3^. Least-squares fitting to our data yields *a* = 2.42 (18), *b* = −2892 (433), and *c* = −32.0 (87).

### Incorporation of hydrogen during core formation

**Fig. 3:**
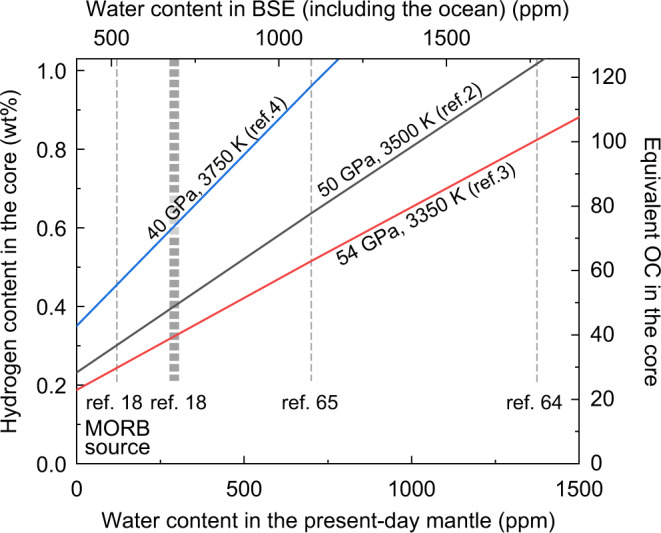
Estimate of hydrogen concentration in the core based on single-stage core formation models. Three solid lines show our estimates of the hydrogen content in the core as a function of residual H_2_O abundance in silicates (= present-day BSE water content) at the labeled *P–T* conditions^2–4^. The BSE water content depends largely on the average mantle abundance (lower horizontal axis)^18,64,65^. Even a modest amount of water in the present-day mantle^18^ and resulting 687 ppm H_2_O in the BSE suggest 0.32–0.61 wt% H in the core. The corresponding ocean mass of water (OC) that includes an equivalent amount of hydrogen is also indicated (right vertical axis).

The partition coefficient only constrains the relative proportion of hydrogen in metal and silicates at equilibrium. In order to estimate the amount of hydrogen that entered metal during core formation, it is necessary to specify the concentration of H_2_O in the silicate magma ocean (MO) with which it equilibrated. If we assume that negligible hydrogen is able to enter the core after formation, and that hydrogen was continuously delivered to the proto-Earth during accretion (as opposed to late), then plausible estimates of the residual amount in the present-day mantle (291 ppm)^18^, crust, oceans, and atmosphere (1.6 × 10^24^ g)^19^, altogether comprising the bulk silicate Earth (BSE) during accretion, gives a value of 687 ppm H_2_O. If the Earth lost 0.1–1 ocean mass of water^20,21^ (OC) by escape of hydrogen to space, this estimate would be considered a lower bound for H_2_O concentrations prevailing in the MO. Acknowledging the many uncertainties in this estimate, we will use 687 ppm H_2_O in the proto-Earth BSE (Fig. 3) in order to examine how much hydrogen could plausibly have been incorporated into the Earth’s core.

We calculated the amount of hydrogen that would be incorporated from silicates into metals based on three different core formation models. First we considered a conceptually simple, single-stage core formation model^2–4^ (see “Methods”), which reconciles the mantle abundances of moderately siderophile elements with entire core–mantle chemical equilibration taking place at a single *P*, *T*, and *f*O_2_ condition, around 50 GPa and 3500 K (Supplementary Fig. 1) and ΔIW = −2.3. While such a model is physically unrealistic, it is nevertheless useful as a simple reference for comparison. With these assumptions, we estimate that a core forged under this scenario would contain 0.32–0.61 wt% H in equilibrium with molten silicate containing 687 ppm H_2_O (Fig. 3 and Supplementary Table 2).

Next we examined a continuous core formation model^5^ (Supplementary Fig. 4) in which core segregation occurred over 1000 accretion events and the impactor core equilibrated with the entire MO at *P–T* conditions prevailing at its base (see “Methods”). We followed a specific model (path 6) given by Badro et al.^5^ for the evolution of *f*O_2_ in metal–silicate equilibration. Considering three different water delivery scenarios, we find that 0.3–0.6 wt% H in the core is necessary to leave a residual budget of ~690 ppm H_2_O in the BSE unless water was delivered only in the last stage of Earth accretion.

**Fig. 4:**
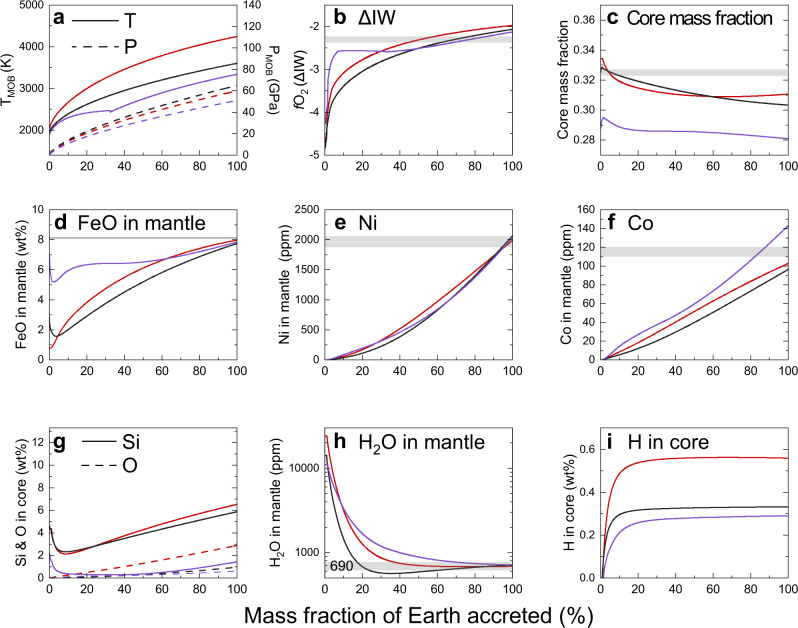
Evolutions during multi-stage core formation. **a** *P* and *T* and **b** *f*O_2_ conditions for metal–silicate equilibrium. **c** Core mass fraction. FeO (**d**), Ni (**e**), Co (**f**), and H_2_O (**h**) in the silicate; and O and Si (**g**) and H (**i**) in the core. Each model, S1 (red), F2 (black), and R3 (purple) in Supplementary Table 4, employed a different combination of *P–T* path (**a**) and partitioning data^2,3,6,57,63^ for elements other than hydrogen (see Supplementary Table 3 for each parameter set). The present Earth values are shown by gray bands.

We also applied a multi-stage core formation model^6^ (Fig. 4 and Supplementary Fig. 5), where metal–silicate partitioning took place by 1000 steps upon accretion of identical impactors having the same water abundance and the metal from each impactor equilibrated only with a limited fraction of silicate melt at the base of the existing MO (see “Methods”). In this model, *f*O_2_ is given as a consequence of the metal–silicate partitioning of H, Ni, Co, O, and Si. We have explored reasonable sets of parameters—impactor size, final (maximum) *P* at the bottom of the MO, impactor’s metal/oxide proportions of Fe and Si, and H_2_O concentration (Supplementary Fig. 6 and Supplementary Table 3), which allow for a remaining ~700 ppm H_2_O in the BSE and yielding the correct core mass and mantle FeO, Ni and Co abundances. These accretion models imply that the core includes 0.27–0.56 wt% H after accretion (Supplementary Table 4).

In summary, our new experimental determination of metal–silicate partitioning of hydrogen suggests 0.3–0.6 wt% H was incorporated into the core, leaving ~700 ppm H_2_O in a magma ocean, unless water was delivered only in the late stage of Earth accretion^22^. We would find more hydrogen in the core if magma oceans contained more water (Fig. 3). According to theoretical calculations^23,24^, a hydrogen-rich outer core is compatible with seismological observations, with 0.3–0.6 wt% H accounting for 30–60% of the density deficit and sound velocity excess of the outer core. A value of 0.3–0.6 wt% H in the core is equivalent to the amount of hydrogen in 37–73 OCs (Fig. 3), and an additional ~2 OCs are included in our assumed BSE (including the ocean itself). Such a large amount of water, corresponding to 0.9–1.7% Earth’s mass, must have been delivered to our planet during accretion. The core is expected to have a lower D/H than equilibrated silicates^25^, some of which may have leaked into the mantle^26^ along with other core-like isotopic signatures^27^. The delivery of a few tens to hundreds times OCs to the growing Earth is supported by some recent planet formation scenarios^28–30^. In addition, any terrestrial planets and moons greater than ~10% of the Earth’s mass may contain >0.15 wt% H in their metallic cores (Supplementary Fig. 5i) if they formed under conditions similar to that of Earth’s formation and water was continuously supplied during accretion.

## Methods

### Starting materials

We used pure iron foil (5N, Mairon-UHP, Toho Zinc Co. Ltd.) and hydrous MORB glass as starting materials. MORB glasses containing about 0.2 and 1.0 wt% H_2_O were synthesized using a piston-cylinder apparatus. The major element composition is similar to the one employed in previous experimental studies^31,32^ (Supplementary Table 5). Water content in the glass was confirmed using SIMS analyses (see below).

### High-pressure and -temperature experiments

Experiments were performed in a laser-heated DAC with flat 300 μm culet diamond anvils. Iron foil (5–7 μm thick) was sandwiched between the hydrous MORB glass powder and loaded into a ~140 µm hole at the center of a pre-indented rhenium gasket. We used the MORB glass with ~1.0 wt% H_2_O, except for run #1 in which ~0.6 wt% H_2_O was added by mixing glass containing 0.2 and 1.0 wt% H_2_O. After loading the sample, the entire DAC was dried in a vacuum oven at 400 K for 3–8 h. As soon as the DAC was taken out of the oven, we immediately started compression.

The sample was heated at high pressure while collecting in situ XRD data at beamline BL10XU, SPring-8. Heating was applied from both sides using two 100 W single-mode Yb fiber lasers (SPI photonics). The laser beam was converted to a flat-top distribution using beam shaping optics (New Focus). The laser spot size was around 40 μm. Heating duration was limited to 3 s to avoid temperature fluctuations that could give rise to complex melting textures. This is sufficient time for each element to diffuse in liquid metal^33^ and silicate^34^. Earlier time-series experiments^32^ on element partitioning in a DAC also showed that partition coefficients did not change after heating for 1–2 s. Temperature was measured using a spectro-radiometric method, and a one-dimensional radial temperature profile was obtained across a laser-heated spot with 1 μm spatial resolution. Experimental temperature was estimated by averaging over the 6 μm span of the X-ray beam. Temperature uncertainty^35^ may be ±5%. Sample pressure was determined from the Raman shift of the diamond anvil^36^ at ambient temperature after heating. We added an additional contribution of thermal pressure that has been estimated to be +2.5 GPa per 1000 K^37^ .

### In situ XRD measurement and the hydrogen content in metal

XRD patterns were collected before/during/after heating in each run. A monochromatic X-ray beam with an energy of ~30 keV was focused to 6 μm area (full-width at half-maximum) on a sample position. The XRD data were collected every 0.22 s during heating on a flat panel detector (Perkin Elmer) (Fig. 1a).

Since hydrogen escapes from solid iron during decompression as it transforms into bcc Fe^10,16^ (Supplementary Fig. 2), hydrogen concentration must be measured at high pressure. The XRD patterns demonstrate that molten iron crystallized FeH_*X*_ and minor FeOOH upon quenching the temperature while maintaining compression (Fig. 1a). The disappearance of a diffuse signal suggests that quenched molten iron fully consisted of crystals even without thermal annealing. Therefore the FeH_*X*_ + minor FeOOH represents the bulk hydrogen concentration in metal. Hydrogen content in molten iron was therefore determined from (1) the proportions of solid FeH_*X*_ and FeOOH in quenched liquid iron and (2) the *X* value in FeH_*X*_ that is calculated from its unit-cell volume at high pressure (Supplementary Table 1). Earlier experiments have demonstrated that thermal annealing of such FeH_*X*_ quench crystals had a relatively small effect on their unit-cell volume; hydrogen content *X* derived from the lattice volume (see below) decreased from 1.21 to 1.14 upon heating to ~1500 K at 65 GPa^17^.

Here, hcp FeH_*X*>1_ and fcc FeH_*X*<1_ were formed in run #4 and the other runs, respectively. Since hydrogen incorporation expands the volume of Fe, hydrogen concentration in FeH_*X*_ can be obtained^38^ as:

3 $$X = (V_{{\mathrm{FeH}}_X} - V_{{\mathrm{Fe}}})/\Delta V_{\mathrm{H}}$$

where $V_{{\mathrm{FeH}}_X}$ is the volume of FeH_*X*_ per Fe atom and Δ*V*_H_ is the volume increase per hydrogen atom^39^, consistent with recent neutron diffraction data^40,41^. The volume of pure Fe is from Dewaele et al.^42^ for hcp and from Tsujino et al.^43^ for fcc. The *X* value was found to be homogeneous in the metal.

The unit-cell volume of ε-FeOOH was similar to that observed in recent high-pressure studies at equivalent pressure^44^. Since FeO was not observed, the proportions of FeH_*X*_ and FeOOH are estimated from the Fe and O contents in quenched liquid iron that were obtained by electron probe micro-analyzer (EPMA) analyses (see below).

### SIMS analysis and the hydrogen content in silicate melt

After recovering the DAC sample at ambient conditions, a cross-section at the center of the laser-heated portion was prepared parallel to the compression/laser-heating axis by using a dual-beam focused ion beam (FIB, Versa 3D^TM^, FEI). Textural observation and preliminary compositional characterization were made with a field-emission-type scanning electron microscope (FE-SEM) and an energy-dispersive X-ray spectrometer (EDS) with a silicon drift detector (Bruker) in the dual-beam FIB system (Supplementary Fig. 3).

Subsequently the hydrogen content in a quenched silicate melt was determined with an isotope microscope system installed at the Hokkaido University, which is composed of CAMECA IMS-1270 SIMS and a two-dimensional ion detector, SCAPS (stacked CMOS-type active pixel sensor)^45^. This system provides quantitative maps of secondary ions emitted from sample surface^46,47^ (Fig. 1b), because the CMOS sensor exhibits a good linear relationship between an output voltage and the number of secondary ions^48^). Therefore, we can quantify the abundance of each element from the intensity map.

Two-dimensional images of ^1^H^+^, ^28^Si^+^, and ^40^Ca^+^ with 0.5–1.2 µm spatial resolution for ^1^H^+^ were collected using the ^16^O^−^ primary beam (13 keV, 37 nA) that was focused to 20–30 μm in diameter and rastered across a 100 μm × 100 μm region on the sample surface. We set the contrast aperture to be 100 μm in diameter with the exit slit fully opened. In order to minimize the effect of adsorbed water on the sample surface, we employed the energy slit to be ±20 eV to select kinetic energy ranges of secondary ions from 80 to 120 eV by loading a sample offset voltage of −100 V. The pressure during measurements was 6.5–8.0 × 10^−8^ Pa. Secondary ion images of ^1^H^+^, ^28^Si^+^, and ^40^Ca were obtained sequentially in the following order; ^28^Si^+^, ^1^H^+^, ^28^Si^+^, ^1^H^+^, ^28^Si^+^, and ^40^Ca^+^. Accumulation time was 500 and 100 s in each image, and two and three images were combined to calculate concentration maps for ^1^H^+^ and ^28^Si^+^, respectively.

Hydrogen concentration in the quenched silicate melt was quantified from the ^1^H/^28^Si intensity ratio using a calibration curve established by three silicate glasses with known H_2_O concentrations (0.00–4.5 wt%)^49^ (Supplementary Fig. 7). The analyses of these standards provide a linear relation between the count and known ^1^H/^28^Si ratios; a correlation coefficient *R*^2^ = 0.996. The detection limit of H_2_O is 15 ppm from its *y*-intercept. These standards were measured before, during, and after the analysis of each DAC sample. In order to reduce statistical errors for each DAC sample, regions of interest (ROIs) were selected on the quenched silicate melt. Combining the ^1^H/^28^Si intensity ratio with the Si content obtained by a field-emission-type electron probe micro-analyzer (FE-EPMA, see below), hydrogen (water) concentration in silicate melt was determined with ±2% to ±7% uncertainty, depending on its abundance (Supplementary Table 1).

### Chemical analysis with FE-EPMA

The major element compositions, except hydrogen, of both quenched liquid metal and silicate melt were determined using an FE-EPMA (JXA-8530F, JEOL) (Supplementary Tables 5 and 6). Analyses were performed with an acceleration voltage of 12 keV and a beam current of 15 nA. For metal, we used an electron beam diameter of 3 μm, Fe, Al_2_O_3_, Ni_2_Si, and Mg as standards, and LIFH (Fe), LDE1H (O), PETJ (Si), and TAP (Al, Mg) as analyzing crystals. For silicate, the standards were SiO_2_, TiO_2_, Al_2_O_3_, Fe_2_O_3_, MgO, CaSiO_3_, NaAlSi_3_O_8_, and KAlSi_3_O_8_, and the analyzing crystals were TAP (Si, Mg, Na), PETJ (Ti, K), TAPH (Al), LIFH (Fe), and PETH (Ca). We employed the EDS analyses of oxygen and silicon only for run #2, because the metal part was sputtered out during SIMS analyses. In addition, the carbon contents in metal were determined separately without carbon-coating using the LDE2 analyzing crystal and Fe_3_C as a standard^17^.

### Activities of elements in hydrogen-rich metal and oxygen fugacity

The hydrogen content *X* in FeH_*X*_ ranged from 0.23 to 1.78 in the present study (Supplementary Table 1). Large *X* significantly decreases the molar fractions of Fe and O in metal. And, it apparently increases the oxygen fugacity $\Delta {\mathrm{IW}} \approx 2\log _{10}\left( {x_{{\mathrm{FeO}}}^{{\mathrm{silicate}}}{\mathrm{/}}x_{{\mathrm{Fe}}}^{{\mathrm{metal}}}} \right)$ and decreases the exchange coefficient $K_{\mathrm{D}}^{\mathrm{O}} = \frac{{x_{{\mathrm{Fe}}}^{{\mathrm{metal}}}x_{\mathrm{O}}^{{\mathrm{metal}}}}}{{x_{{\mathrm{FeO}}}^{{\mathrm{silicate}}}}}$ for the reaction FeO^silicate^ = Fe^metal^ + O^metal^.

We obtain the $K_{\mathrm{D}}^{\mathrm{O}}$ values from this study using molar fractions of iron and oxygen in metal, with and without considering the presence of hydrogen and carbon. They are compared with the results of Fischer et al.^3^ that were obtained in the absence of hydrogen and carbon (Supplementary Fig. 8). The present $K_{\mathrm{D}}^{\mathrm{O}}$ values that do not take hydrogen and carbon into account are closer to their data, suggesting that hydrogen and carbon do not have colligative properties in iron solvent. It is likely because the incorporation of small H and C atoms in liquid Fe is interstitial rather than substitutional unlike other larger atoms^23^. Therefore, in this study, we approximate the activity of element *i* in metal as:

4 $$x_i^\prime = \frac{{N_i}}{{\mathop {\sum}\nolimits_{k \ne {\mathrm{H,C}}} {N_k} }}$$

Note that this formulation has been conventionally used in solid metal-hydrogen systems^50^. The slope on a plot log_10_*D* vs. ΔIW obtained with this formulation indicates a valence state of 0.999 for hydrogen in the silicate melt (Supplementary Fig. 9), which supports the validity of the present estimate of the activities of elements in metal and silicate. Our experiments give log_10_$K_{\mathrm{D}}^{\mathrm{O}}$ = *a* + *b*/*T*, where *a* = 0.37 (18) and *b* = −1535 (687).

### Single-stage core formation model

Single-stage core formation models^2–4^ assume the entire core and mantle equilibrate at a single *P*, *T*, and *f*O_2_ condition. We employed three different *P–T* conditions at fixed *f*O_2_ (ΔIW = −2.3) that is compatible with the FeO content of the mantle (Supplementary Table 2), which have been proposed on the basis of different sets of partitioning data for siderophile elements^2–4^. The present experiments demonstrate that hydrogen is strongly siderophile under these conditions; the metal/silicate partition coefficient $D_{\mathrm{H}}^ \ast$ ranges from 38 to 70 (Supplementary Fig. 1) (see its caption for $D_{\mathrm{H}}^ \ast$ that is defined for simplicity). Previous studies^2–4^ have already calculated the partitioning of O and Si. We assume that the presence of H and H_2_O does not change the partitioning of siderophile elements^51,52^, O (Supplementary Fig. 8) and Si.

Our calculations give 0.32–0.61 wt% H in the core when equilibrated with a KLB-1 pyrolitic melt^53^ containing 687 ppm H_2_O (Fig. 3). The resulting core also includes 0 (assumed)–2.5 wt% O and 4.5–13.3 wt% Si, in addition to 0.32–0.61 wt% H and likely 2.0 wt% S that is inferred from cosmo-/geochemical arguments^54^ (Supplementary Table 2). This liquid is less dense than the present outer core^24,55^, possibly suggesting that it later become depleted in Si + O following SiO_2_ crystallization^56^.

### Continuous core formation model

The continuous core formation model considers that the Earth accreted incrementally and the metal in each impactor chemically equilibrated with the entire magma ocean (MO). The *P–T* conditions are assumed to correspond to the base of the MO^4,5^. While Wade and Wood^4^ argued that the oxidation state for core segregation was initially reductive and became progressively more oxidative to the current state (ΔIW = −2.3), Badro et al.^5^ demonstrated that starting with oxidative conditions can better explain the mantle abundances of moderately siderophile elements on the basis of metal–silicate partitioning experiments at higher pressures^57^.

We performed 1000-step calculations of metal–silicate partitioning of hydrogen at evolving *P*, *T*, and *f*O_2_ conditions following path 6 in Badro et al.^5^; for *i*^th^ step, *P*_MOB,*i*_ = *P*_final_ × (*i*/1000)^2/3^ (*P*_final_ = 62 GPa) and *T*_MOB1,*i*_ = 2022 + 54.21*P*_MOB,*i*_ − 0.34 $P_{{\mathrm{MOB}},i}^2$ + 9.0747 × 10^−4^ $P_{{\mathrm{MOB}},i}^3$ (K) (Supplementary Fig. 1), which explains the mantle abundances of Ni, Co, V, and Cr as well as FeO. Details are found in Dauphas^58^. Each impactor possesses 32.5 wt% metal (same as the present-day Earth). Silicate composition is pyrolitic^59^ except for FeO that is controlled by *f*O_2_ in the model. Evolution of the Si and O abundances in the core are shown in Fig. S2 in Badro et al.^5^. The effects of H_2_O and H on metal/silicate partitioning of the other elements are not considered (see^51,52^ and Supplementary Fig. 8). According to Iizuka-Oku et al.^10^ and Fukai and Suzuki^16^, no hydrogen is partitioned into metal when *P*_MOB,*i*_ < 3 GPa.

Three different scenarios were considered for the delivery of water: (1) constant, (2) linearly increases, and (3) none but the last seven impactors (Supplementary Fig. 4a). For each scenario, we have examined hydrogen concentrations in the core when ~690 ppm H_2_O remains in the silicate after accretion (Supplementary Fig. 4b). For scenario 1, hydrogen concentration in the core is almost constant at 0.60 wt% during accretion (Supplementary Fig. 4c). While 0.30 wt% H is found in the core with scenario 2, only 25 ppm is present in scenario 3. It is possible that water derives predominantly from a late veneer after core formation^22^, leading to the minimal incorporation of hydrogen into the core.

### Multi-stage core formation model

We also examined the multi-stage core formation model, in which core segregation occurred by multiple steps upon accretional impacts and each time impactor core metal equilibrated with a limited portion of silicate in the existing MO at the *P–T* condition of its base^6,60^. While Rubie et al.^6^ assumed that hydrogen is not siderophile and employed *D*_H_ = 0.05–0.5, we apply *D*_H_ > 34 above 3 GPa when following the same *P–T* evolution for metal–silicate equilibration in this study.

Our calculations followed the concepts of Rubie et al.^60^ except that impactor size and composition does not change during accretion. Upon each impact on the proto-Earth, an impactor core sinks in a descending plume, which expands with increasing depth as more silicate melt is turbulently entrained (see Fig. 2 in Deguen et al.^61^). The equilibrium efficiency between impactor metal and the proto-Earth’s silicate is controlled by *Γ* at each step, which is the ratio of the mass of equilibrated silicate over the mass of each impactor core. We performed 1000-step calculations of metal–silicate partitioning, with *N* (1–10) impactors for each step.

At *i*^th^ step, *Γ*_*i*_ is obtained as follows. The volume fraction of metal (*φ*_metal,*i*_) in such metal + silicate composite plume when it reaches the bottom of the MO is given by Deguen et al.^61^,

5 $$\varphi _{{\mathrm{metal}},i} = \left( {\frac{{r_{{\mathrm{imp}}\,{\mathrm{core}}}}}{{r_{{\mathrm{plume}}}}}} \right)^3 = \left( {1 + \frac{{\alpha Z_i}}{{r_{{\mathrm{imp}}\,{\mathrm{core}}}}}} \right)^{ - 3}$$

in which α = 0.25, *Z*_*i*_ is depth of the MO, and *r*_imp core_ is the radius of impactor’s core. *r*_imp core_ is formulated as:

6 $$r_{{\mathrm{imp}}\,{\mathrm{core}}} = \left[ {\frac{{m_{{\mathrm{imp}}} - \frac{4}{3}\pi \times r_{{\mathrm{imp}}}^3 \times \rho _{{\mathrm{imp}}\,{\mathrm{silicate}}}}}{{\frac{4}{3}\pi \times \left( {\rho _{{\mathrm{imp}}\,{\mathrm{core}}} - \rho _{{\mathrm{imp}}\,{\mathrm{silicate}}}} \right)}}} \right]^{1/3}$$

with impactor’s entire mass *m*_imp_ (=1/1000/*N* × Earth’s mass), silicate and core densities *ρ*_imp silicate_ = (1.446/1000/*N* + 3) × 10^3^ (kg m^−3^) and *ρ*_imp core_ = 2.5 × *ρ*_imp silicate_^6^ and entire radius *r*_imp_ of the impactor,

7 $$r_{{\mathrm{imp}}} = \left[ {\frac{{m_{{\mathrm{imp}}\,{\mathrm{silicate}}} + 0.4\,m_{{\mathrm{imp}}\,{\mathrm{core}}}}}{{\frac{4}{3}\pi \times \rho _{{\mathrm{imp}}\,{\mathrm{silicate}}}}}} \right]^{1/3}$$

Both the mass of impactor silicate and core, *m*_imp silicate_ and *m*_imp core_, are obtained when Φ_Fe_, Φ_Si_ (the proportions of metallic Fe and Si with respect to all Fe and Si) and H_2_O concentration of the impactor are given. *r*_imp_ is found, for example, to be 408 km when Φ_Fe_ = 0.99, Φ_Si_ = 0.17, H_2_O=1.1 wt% and *N* = 1 (Supplementary Table 3). It could be argued that the average impactor size during Earth accretion was smaller (*N* > 1) considering the biggest asteroid Ceres is 473 km in radius.

*Z*_*i*_, the depth of the MO at step *i*, is given as *r*_pEarth,*i*_ − *r*_MOB,*i*_. *r*_pEarth,*i*_ is the radius of the entire embryo (proto-Earth), which is calculated using Eq. 7 with its silicate density *ρ*_pEarth silicate,*i*_ = (1.446 × *i* /1000 + 3) × 10^3^ (kg m^−3^) and the mass of its silicate and core, *m*_pEarth silicate,*i*_ and *m*_pEarth core,*i*_, that are calculated at each step. *r*_MOB,*i*_, the radius of the bottom of the MO, was obtained from the following equations with corresponding pressure *P*_MOB,*i*_ = *P*_*i*_ (GPa) for metal–silicate equilibrium^5,62^,

8 $$P_{{\mathrm{MOB}},i} \times 10^9 	= P_{{\mathrm{final}}} \times \left( {\frac{i}{{1000}}} \right)^{2/3} \times 10^9\\ 	= \frac{4}{3}\pi G\rho _{{\mathrm{pEarth}}\,{\mathrm{silicate}},\,i}\ r_{{\mathrm{MOB}},i}^3\left( {\rho _{{\mathrm{pEarth}}\,{\mathrm{core}},\,i} - \rho _{{\mathrm{pEarth}}\,{\mathrm{silicate}},\,i}} \right)\left( {\frac{1}{{r_{{\mathrm{MOB}},i}}} - \frac{1}{{r_{{\mathrm{pEarth}},i}}}} \right)\\ 	\ \ + \frac{2}{3}\pi G\rho _{{\mathrm{pEarth}}\,{\mathrm{silicate}},\,i}^2\left( {r_{{\mathrm{pEarth}},i}^2 - r_{{\mathrm{MOB}},i}^2}\right) $$

with gravitational constant *G*. For example, in an embryo with 1/10 Earth’s mass and a radius of ~3200 km, the depths of the core–mantle boundary and the bottom of the MO (*Z*_100_) are ~1500 and ~900 km, respectively, depending on Φ_Fe_ and Φ_Si_.

Finally *Γ*_*i*_ is obtained as:

9 $$\Gamma _i = \frac{{m_{{\mathrm{equilibrated}}\,{\mathrm{silicate}}}^\prime }}{{m_{{\mathrm{imp}}\,{\mathrm{core}}}^\prime }} = \frac{{\frac{4}{3}\pi{}r_{{\mathrm{imp}}\,{\mathrm{core}}}^{\prime 3} \times \left( {\frac{1}{{\varphi _{{\mathrm{metal}},{i}}}} - 1} \right) \times \rho{}_{{\mathrm{pEarth}}\,{\mathrm{silicate}},{i}}}}{{m_{{\mathrm{imp}}\,{\mathrm{core}}}^\prime }}$$

with *φ*_metal,*i*_ (Eq. 5) and the radius *r*′_imp core_ and mass *m*′_imp core_ of combined *N* impactors accreting in each 1/1000 step of metal–silicate partitioning. Eq. 9 indicates that *Γ*_*i*_ is larger (impactor core reacts with more silicate) when *φ*_metal,*i*_ (namely, impactor size) is smaller. In addition, we considered that the impactor’s silicate was preferentially involved in the chemical reaction with the impactor metal in the MO (Supplementary Fig. 10).

We have calculated the partitioning of H, Ni, Co, O, and Si between the impactor metal and equilibrated silicate (whose mass is obtained by *Γ*_*i*_) under *P*_MOB,*i*_ = *P*_final_ × (*i*/1000)^2/3^ and three different *T* at each step (Supplementary Fig. 1):

1. *T*_MOB1,*i*_ = (same as that for the continuous core formation model above).
2. *T*_MOB2,*i*_ = 1940 × (*P*_MOB,*i *_/29 + 1)^1/1.9^.
3. $$T_{{\mathrm{MOB3}},i}= \left\{\begin{array}{*{20}{l}}1874 + 55.43P_{{\mathrm{MOB}},i}- 1.74P_{{\mathrm{MOB}},i}^{2} + 0.0193P_{{\mathrm{MOB}},i}^{3 } (< 24 {\mathrm{GPa}}) \\ 1249 + 58.28P_{{\mathrm{MOB}},i}- 0.395P_{{\mathrm{MOB}},i}^{2} + 0.0011P_{{\mathrm{MOB}},i}^{3 } (\ge \,24 \,{\mathrm{GPa}})\end{array}\right.$$

For each of the three *T*_MOB_ profiles, three different sets of metal–silicate partitioning data for Ni, Co, O, and Si^2,3,6,57,63^ were applied. Each impactor had a CI chondritic-like composition for refractory elements (see Table S1 in Rubie et al.^6^). The evolution of *f*O_2_ was calculated as $\Delta {\mathrm{IW}} \approx - 2\log _{10}\frac{{x\prime _{{\mathrm{Fe}}}^{{\mathrm{metal}}}}}{{x_{{\mathrm{FeO}}}^{{\mathrm{silicate}}}}}$ (molar fraction *x*′ does not consider H in metal, see above) at each step, which is the consequences of the incorporation of H, Si, and O into core-forming metal and of Ni, Co and Fe into silicate. We did not consider the partitioning of S into silicate nor that of Al, Mg, and Ca into metal. We assumed that metal–silicate partitioning was not affected by the presence of H and H_2_O (see^51,52^ and Supplementary Fig. 8).

We have explored parameter sets of *N*, *P*_final_, and impactor’s Φ_Fe_, Φ_Si_, and H_2_O concentration, which explain the residual silicate abundances of ~700 ppm H_2_O, ~8.1 wt% FeO, ~2000 ppm Ni, and ~100 ppm Co^59^ (Fig. 4 and Supplementary Fig. 5). The mass fraction of the core, containing ~2 wt% S^54^ in addition to H, Ni, Co, O, and Si, was targeted to be 0.325. Supplementary Fig. 6 shows parameters search maps for reductive, moderate and oxidative impactor cases. With *N* = 3, *T*_MOB1_ and metal–silicate partitioning data reported by Siebert et al.^2,57^, homogeneous accretions of reductive but water-bearing impactors best explain the Earth’s composition (the evolution during accretion is given in Supplementary Data 1). We found a reasonable parameter set for each combination of three different *P–T* paths and three different sets of partitioning data^2,3,6,57,63^ as listed in Supplementary Table 3. These calculations show that hydrogen concentration ranges from 0.27 to 0.56 wt% in the Earth’s core (Supplementary Table 4). The evolutions of *P*, *T*, and *f*O_2_ conditions for metal–silicate equilibrium, core mass fraction, the FeO, Ni, Co, and H_2_O contents in the magma ocean, and the O, Si, and H abundances in the core are illustrated in Fig. 4 and Supplementary Fig. 5.

### Pressure and temperature effects on hydrogen partitioning using previous low-pressure data along with the present results

We also estimated the effects of *P* and *T* on metal–silicate partitioning of hydrogen using earlier low-pressure multi-anvil experimental data^12^ along with the present results. The exchange coefficient *K*_D_ is written as

10 $${\mathrm{log}}_{10}K_{\mathrm{D}} = 0.692\left( {986} \right) - 4590\left( {1690} \right)/T\left( {\mathrm{K}} \right) + 141\left( {43} \right) \,\times P\left( {{\mathrm{GPa}}} \right)/T\left( {\mathrm{K}} \right)$$

They are compared with those obtained only from our data in Supplementary Fig. 11.

With such parameters, we have additionally calculated the amount of hydrogen incorporated into the core on the basis of a multi-stage core formation model that is similar to model #S1 except for the *K*_D_ value (Supplementary Fig. 12, and Supplementary Tables 3 and 4). The results show 0.34 wt% H in the core, consistent with those obtained by other simulations.

## Supplementary information

Supplementary Information

Description of Additional Supplementary Files

Supplementary Data 1

## Data Availability

The data supporting the main findings of this study are available in the paper and it’s Supplementary Information. Any additional data can be available from the corresponding authors upon reasonable request.
