# Supplementary material for: Experimental evidence for hydrogen incorporation into Earth’s core
Source: Nat Commun. 2021 May 11;12:2588. doi: 10.1038/s41467-021-22035-0 (PMC8113257; doi:10.1038/s41467-021-22035-0)
Supplement: Supplementary file 1 — Supplementary Information [file 41467_2021_22035_MOESM1_ESM.pdf]

Supplementary Information for

Experimental evidence for hydrogen incorporation into Earth's core

by Tagawa *et al*.

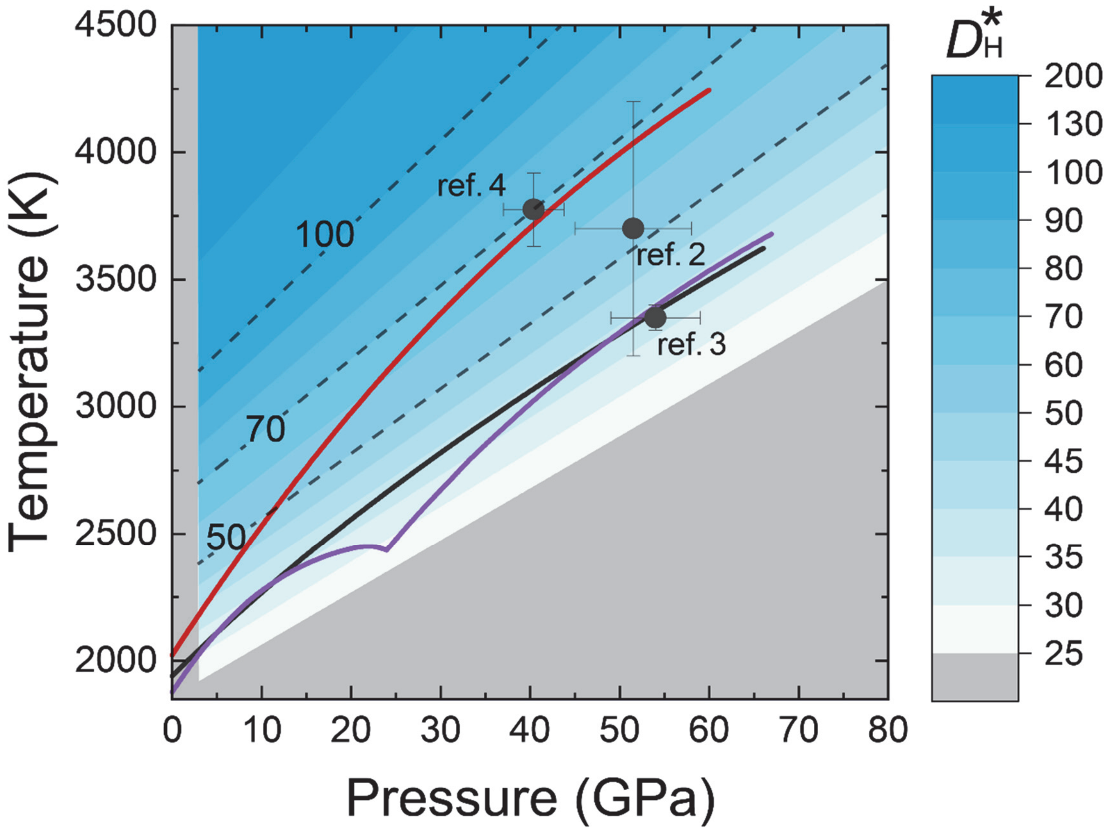

Supplementary Fig. 1 *P-T* conditions for core formation and the metal/silicate partition coefficient of hydrogen D_H^*. The *P-T* conditions for metal-silicate equilibrium based on single-stage core formation models[2–4] are shown by black circles. The *P-T* evolutions in the present continuous and multi-stage core formation models are from earlier modellings; T_{MOB1} (red) adopted in ref. 5, T_{MOB2} (black) in ref. 3 and T_{MOB3} (purple) in refs. 6 & 60. D_H^* is defined for simplicity as $c_H^{*core}/c_H^{silicate}$, in which c_H^{*core} represents hydrogen concentration in iron by weight, without considering other elements so that D_H^* does not depend on the incorporation of O and Si whose amounts change with *P* and *T*. D_H^* at ΔIW = -2.3 is greater than 29 along *P-T* paths considered in any previous core formation models except below ~3 GPa where hydrogen is not partitioned into iron[10,16].

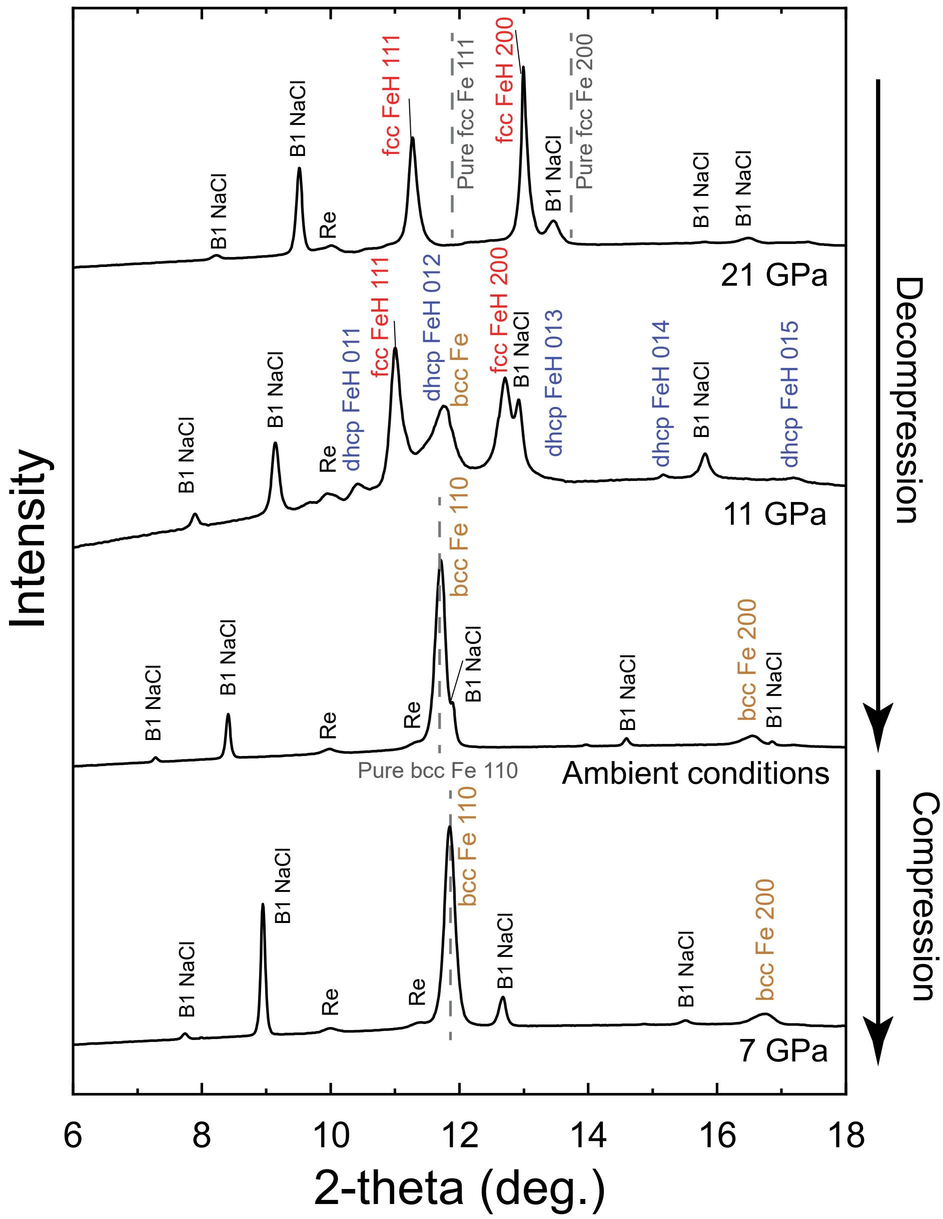

Supplementary Fig. 2 Changes in XRD patterns upon decompression from 21 GPa to ambient pressure and subsequent re-compression to 7 GPa. The FeH_X (X=1.0) sample was sandwiched between the NaCl layers (pressure medium) in a DAC. The lattice volume of the sample was larger than that of pure Fe at 21 GPa but became identical to it after decompression to 1 bar, indicating loss of hydrogen from iron lattice. Upon subsequent re-compression, dhcp FeH was not formed, suggesting that hydrogen did not remain within metal but escaped from the sample.

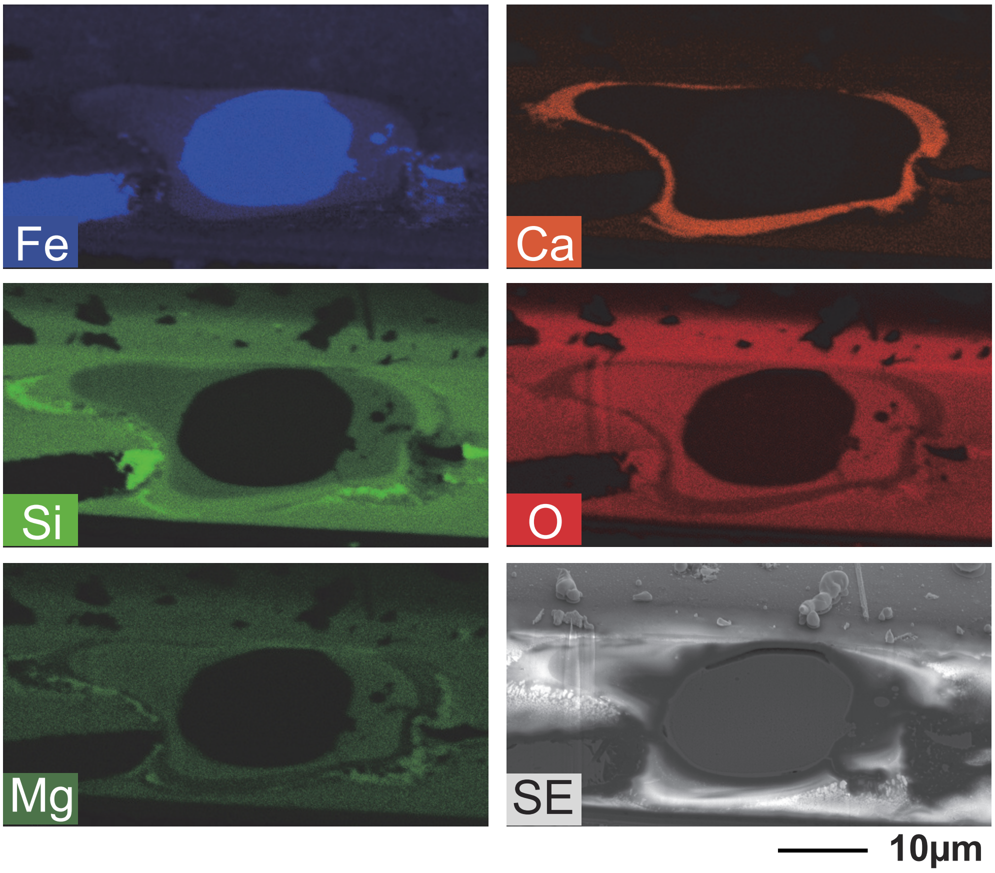

Supplementary Fig. 3 Sample cross-section for run #1. X-ray maps for Fe, Ca, Si, O and Mg and scanning electron microscope image.

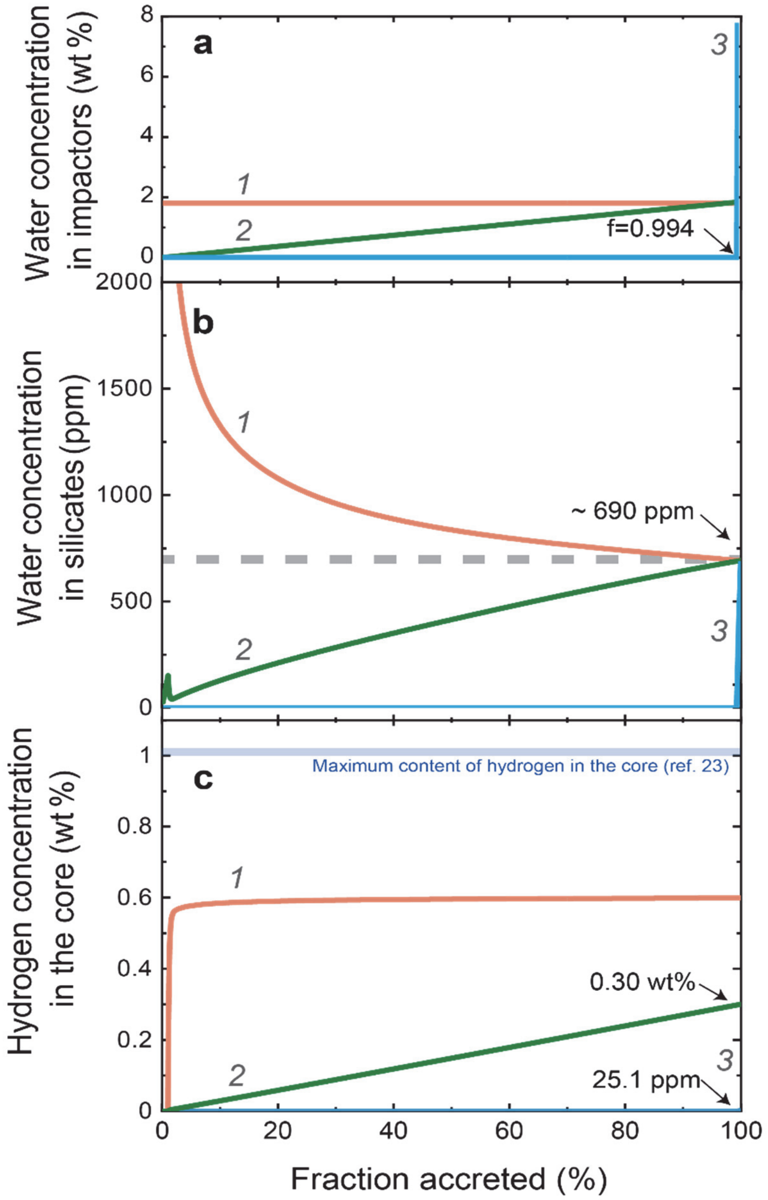

Supplementary Fig. 4 Delivery of water and evolution of H_2O in silicates and H in the core based on continuous core formation model. Evolutions in water concentration in impactors (**a**) and equilibrated silicates (**b**) and in hydrogen content in the core (**c**). Calculations were performed along path 6 in Badro *et al.*[5] with three different water delivery scenarios illustrated in **a**; constant (orange), linear increase (green) and none except the last seven impactors (blue).

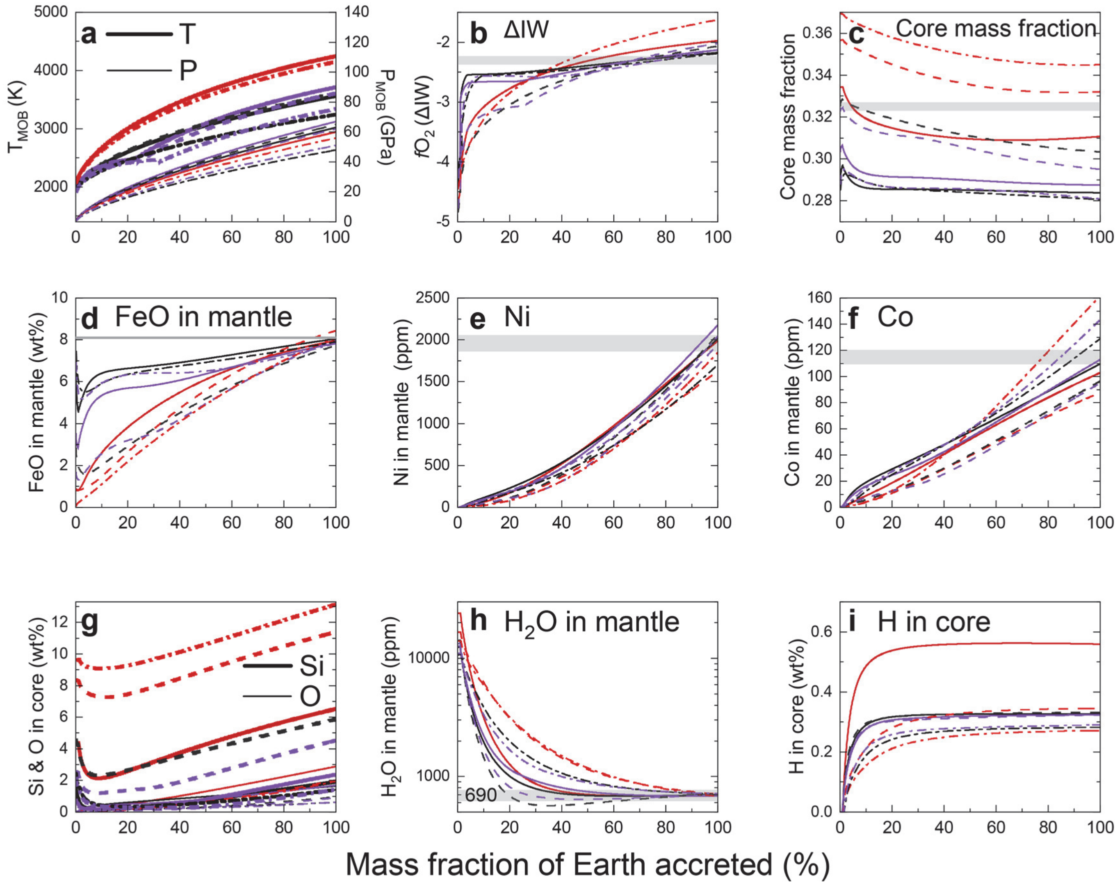

Supplementary Fig. 5 Evolution during multi-stage core formation. (**a**, **b**) *P* & *T* (**a**) and *f*O2 (**b**) conditions for metal-silicate equilibrium; (**c**) core mass fraction; (**d**–**i**) FeO (**d**), Ni (**e**), Co (**f**) and H_2O (**h**) in the silicate, and O & Si (**g**) and H (**i**) in the core. Calculations were made for nine different combinations of partitioning data (solid curves[2,57], S1–S3; dotted curves[3], F1–F3; dash-dotted curve[6], R1–R3) and *P*-*T* path (red, T_{MOB1}; black, T_{MOB2}; purple, T_{MOB3}) (Supplementary Table 4). See Supplementary Table 3 for each parameter set.

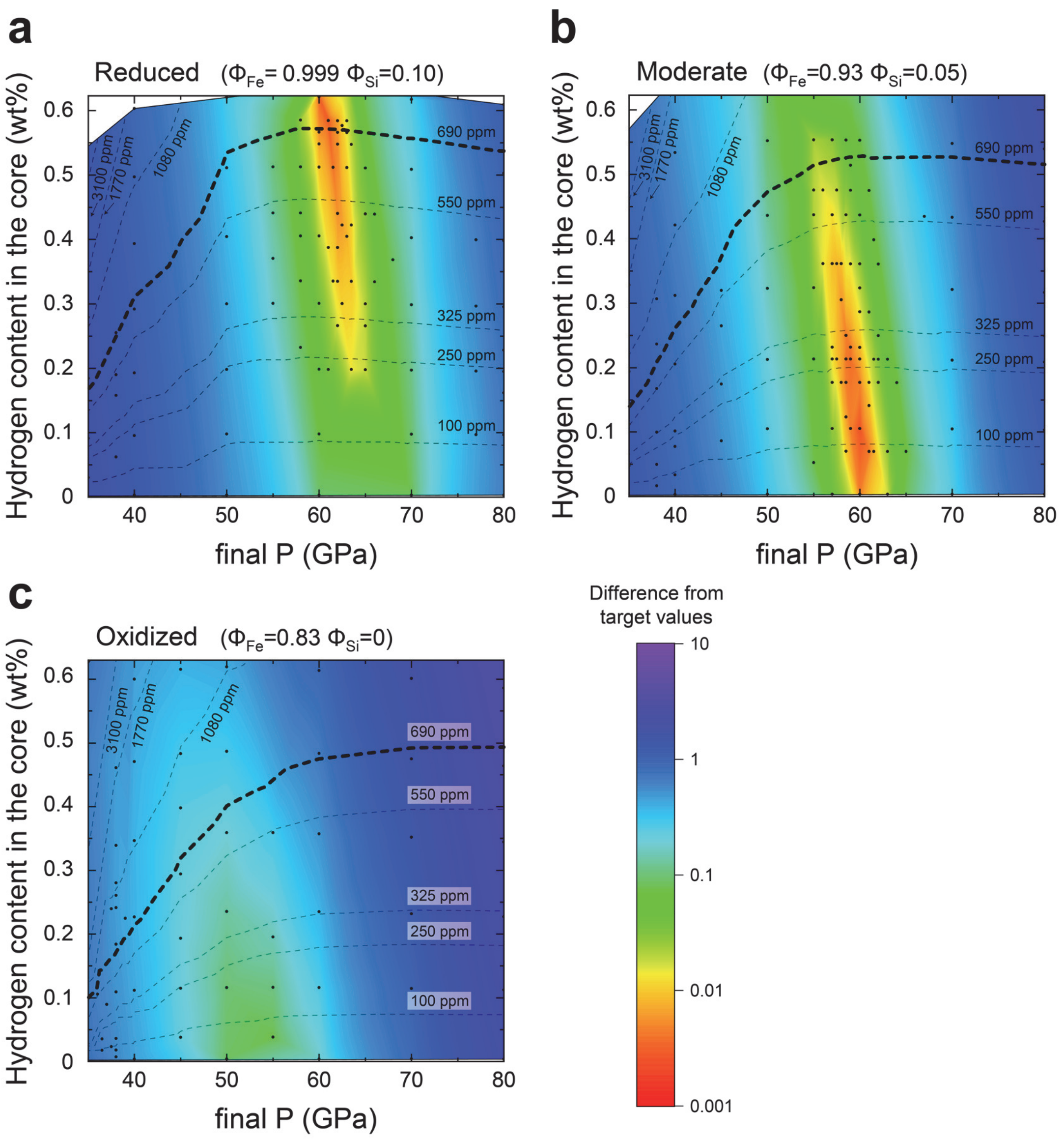

Supplementary Fig. 6 Parameter search maps for reductive, moderate and oxidative impactors. The impactor's Φ_{Fe} and Φ_{Si} values in **a** and **c** are from homogeneous accretion models by Rubie *et al.*[60] (those in **b** are in between). Note that they are different from those of best model parameters sets listed in Supplementary Table 3. Colors indicate the difference from target values, which is defined as Σ((calculated value)/(present Earth value)-1)^2 for core mass fraction and mantle concentrations of FeO, Ni and Co. Dots indicate calculation points. Bold broken lines show the target of leaving ~690 ppm H_2O in silicates after core formation.

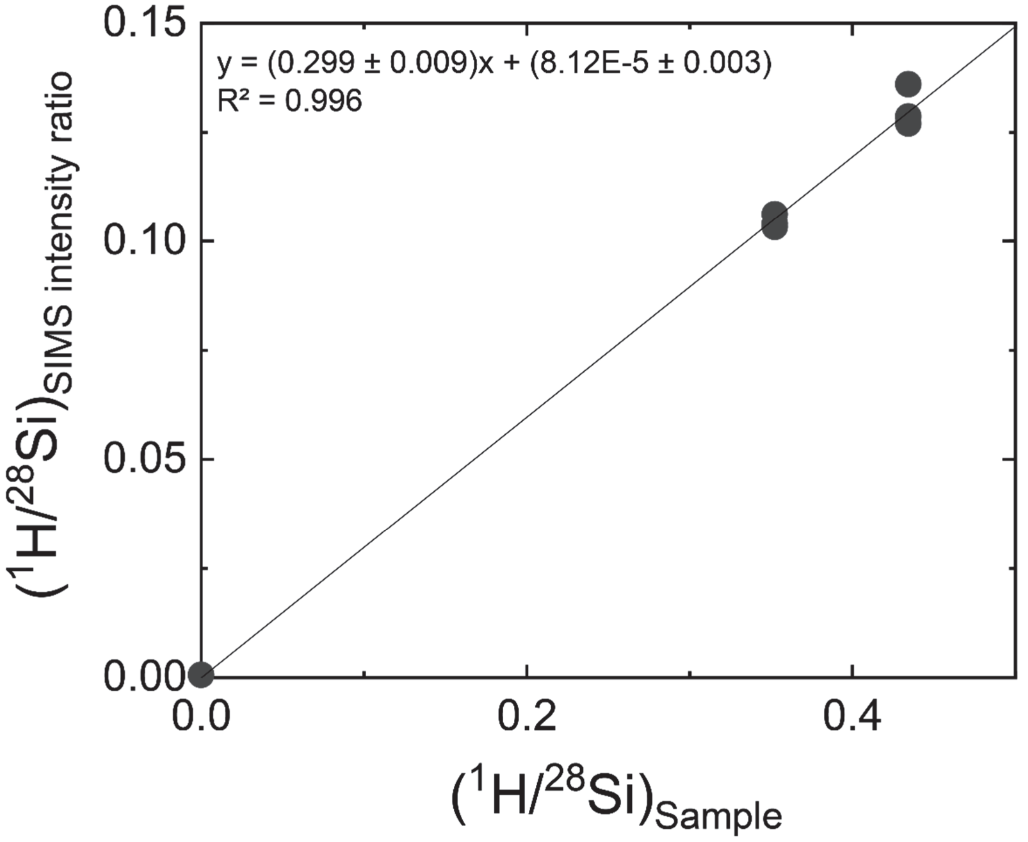

Supplementary Fig. 7 Calibration curve for the SIMS analysis of $^{1}H/^{28}Si$ based on standard glasses.

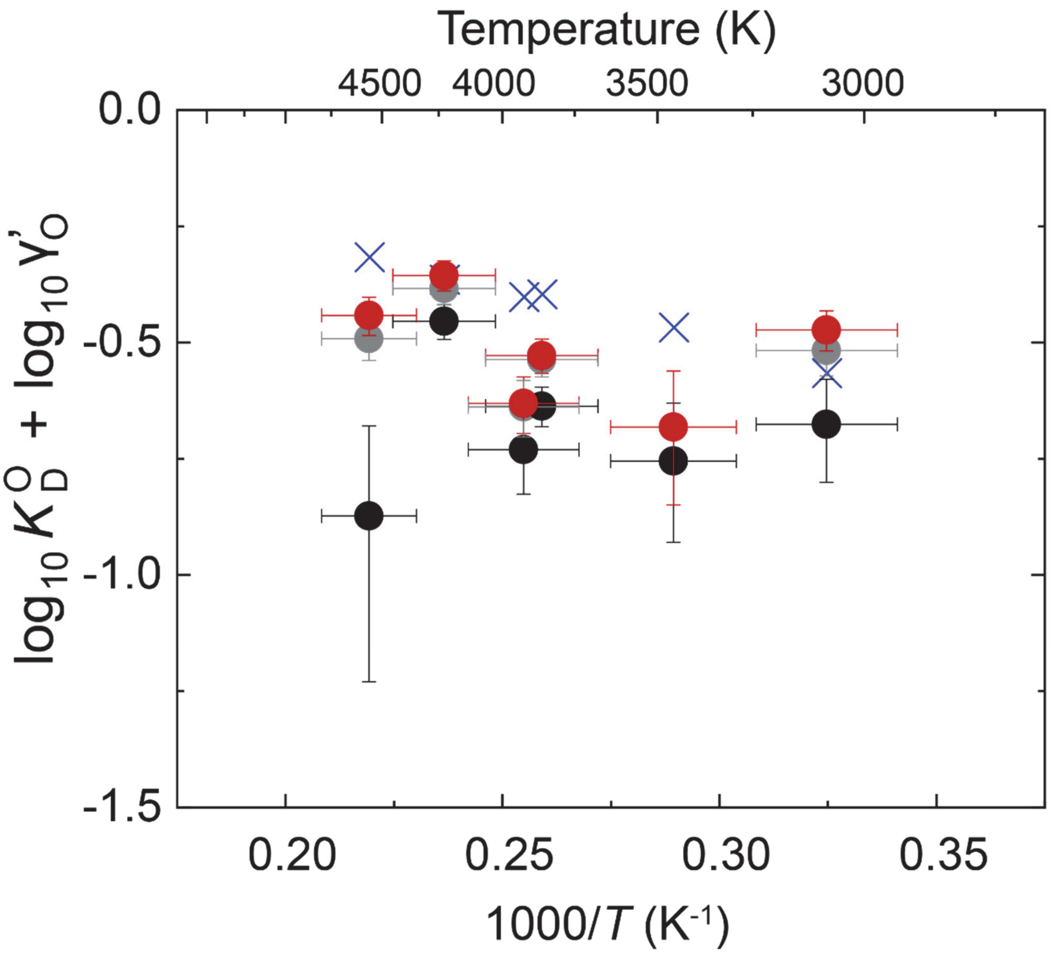

Supplementary Fig. 8 K_D^O with and without considering the presence of hydrogen and carbon for activity estimates. Black and grey circles are based on molar fractions obtained including hydrogen and carbon, respectively, along with silicon and oxygen in liquid iron, while red circles do not include hydrogen nor carbon to obtain the K_D^O values. Crosses show earlier results obtained in the H-free system[3].

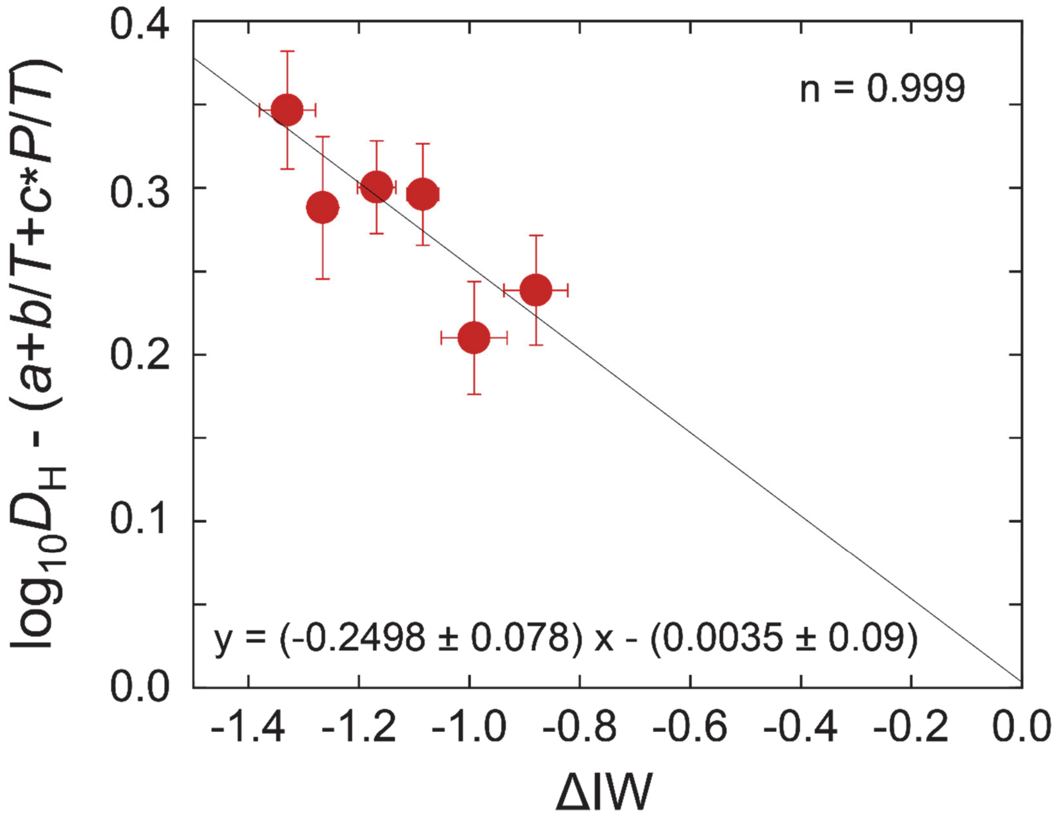

Supplementary Fig. 9 Partition coefficient *D* of hydrogen (molar basis) as a function of oxygen fugacity relative to the IW buffer. The difference in experimental *P* and *T* condition is corrected by the $a + b/T + cP/T$ term. The slope of this plot indicates the valence state (n) of hydrogen in silicate melt to be 0.999.

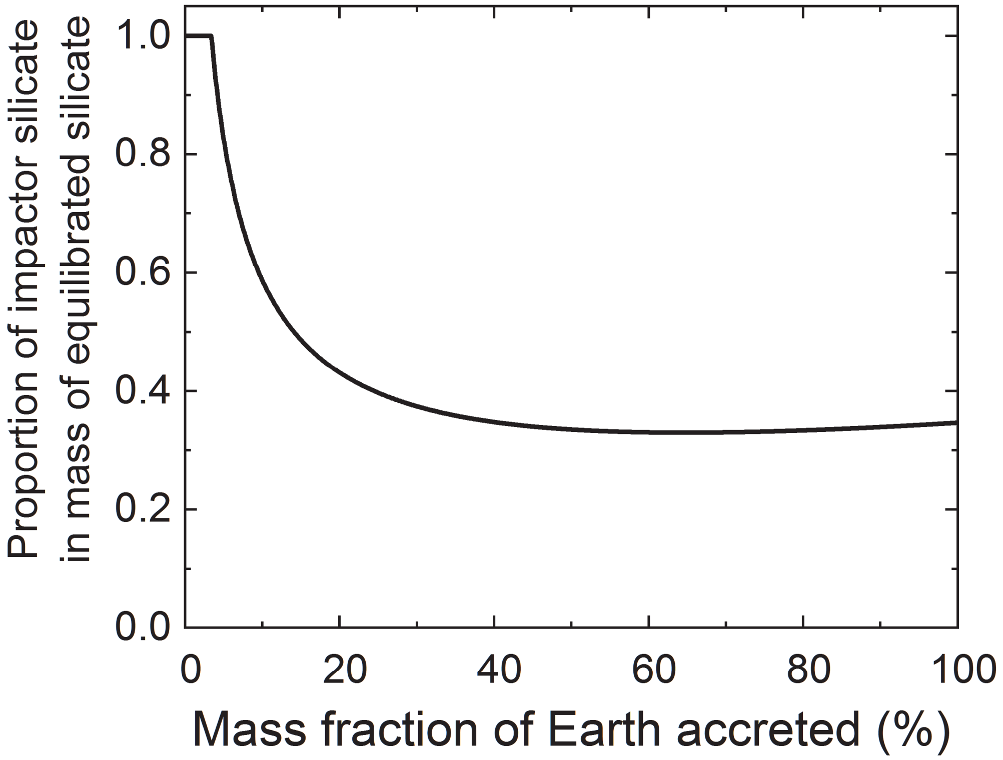

Supplementary Fig. 10 Change in the proportion of impactor silicate in silicate with which impactor core equilibrated. The result for model S1 in Supplementary Tables 3 and 4 is shown.

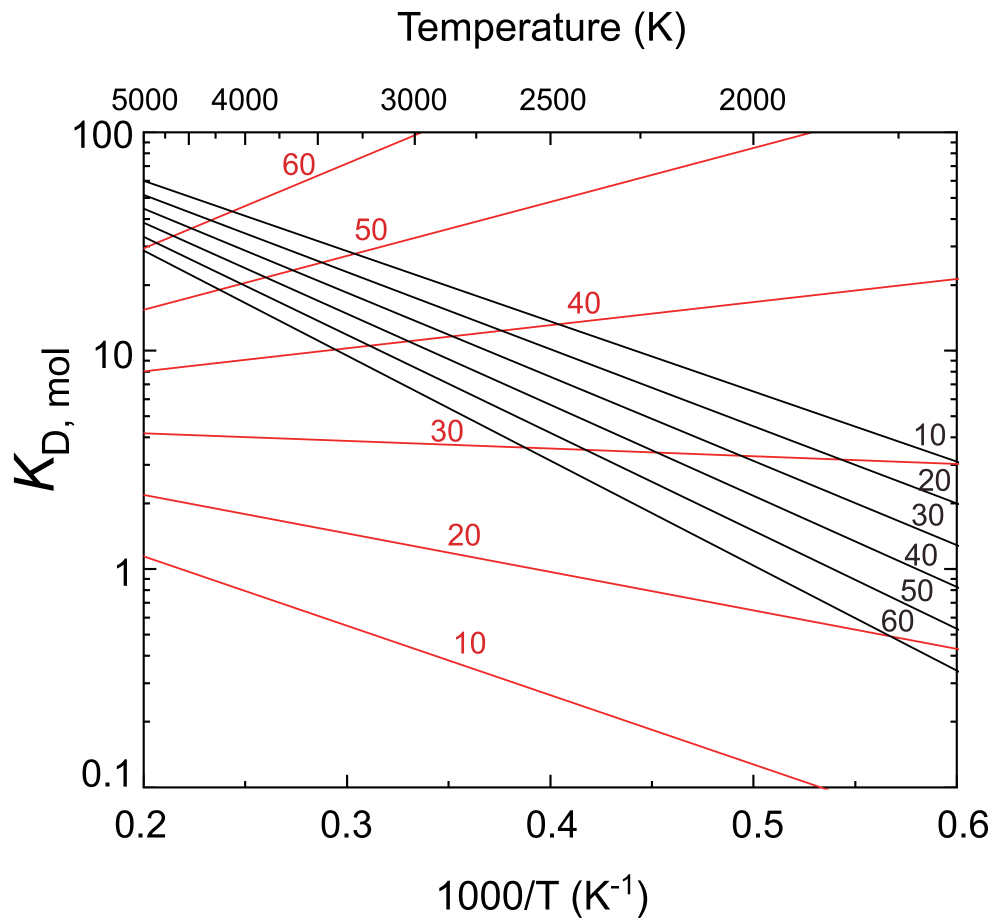

Supplementary Fig. 11 Comparison of exchange coefficient K_D between those obtained only from the present experiments (black lines) and from earlier low-pressure experiments[12] combined with this study (red lines). The numbers given to each datum point and regression line indicate pressure conditions.

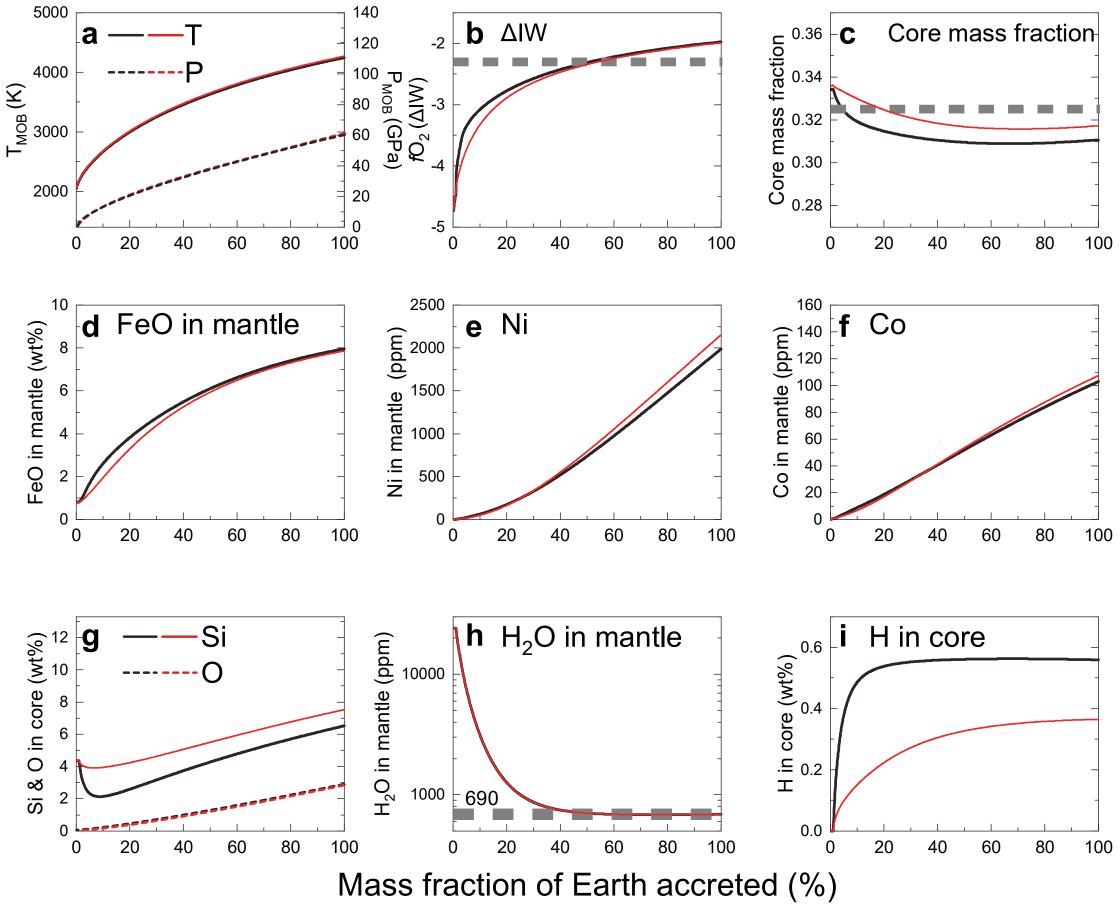

Supplementary Fig. 12 Changes in evolution during multi-stage core formation with different K_D for hydrogen. Similar to Supplementary Fig. 5. Black (model #S1) and red curves (model #S1') show results with K_D from Eqs. 2 and 10, respectively. Other model parameters are given in Supplementary Table 3.

Supplementary Table 1 Summary of the present experiments

Run #	#1	#2	#3	#4	#5	#6
P (GPa)	46(5)	48(5)	57(6)	60(6)	47(5)	30(3)
T (K)	3920 (200)	3450 (170)	3860 (190)	4560 (230)	4230 (210)	3080 (150)
H in metal (ppm wt)	9390 (690)	9030 (690)	9280 (630)	26160 (1600)	5320 (380)	8020 (640)
H in silicate (ppm wt)	201(3)	309(5)	233(5)	466(9)	94(6)	219(4)
D_H	47(4)	29(2)	40(3)	56(4)	57(6)	37(3)
fO_2 (ΔIW)	-0.88	-0.99	-1.08	-1.17	-1.27	-1.33
Structure of FeH_X	fcc	fcc	fcc	hcp	fcc	fcc
Lattice volume of FeH_X (Å^3)	42.84(1)	43.26(11)	41.59(4)	25.14(65)	40.84(3)	44.60(4)
Hydrogen content X	0.52	0.52	0.52	1.78	0.23	0.43
ε-FeOOH proportion*	0.19	0.15	0.18	0.06	0.13	0.07
Area of ROI in SIMS analysis (μm^2)	120	160	80	110	60	80
$\log_{10}K_D^H$	1.32(6)	1.10(6)	1.22(5)	1.37(5)	1.35(6)	1.18(6)

Numbers in parentheses indicate uncertainty in the last digits. Errors in hydrogen concentration in metal derive mainly from the uncertainty in ΔV_H (Eq. 3) in addition to those in the volume of FeH_X and the proportion of FeOOH.

*Defined as $\frac{x_{\varepsilon\text{-FeOOH}}}{x_{\varepsilon\text{-FeOOH}}+x_{\mathrm{FeH_X}}}$

Supplementary Table 2 Conditions and consequences of single-stage core formation

	Conditions			Core composition (wt%)				Contribution to CDD*			
	P (GPa)	*T* (K)	ΔIW	S	Si	O	H	S	Si	O	H
ref. 2	50	3500	-2.3	2.0	4.5	2.5	0.4	14%	47%	29%	41%
ref. 3	54	3350	-2.3	2.0	7.9	1.5	0.3	14%	83%	17%	33%
ref. 4	40	3750	-2.3	2.0	13.3	-	0.6	14%	139%	-	62%

*The outer core density deficit (CDD) is explained by 13.9 wt% S, 9.6 wt% Si, 8.7wt% O or 1.0 wt% H according to Umemoto & Hirose[24].

Supplementary Table 3 Parameter sets for multi-stage core formation models

Model #		S1	S1'*	S2	S3	F1	F2	F3-1	F3-2	R1	R2	R3
Partitioning data		refs. 2, 57				ref. 3				ref. 6		
N		3	3	3	2	1	3	3	10	1	3	3
final P (GPa)		60	60	63	67	60	65	62	61	56	48	51
T		T_{MOB1}	T_{MOB1}	T_{MOB2}	T_{MOB3}	T_{MOB1}	T_{MOB2}	T_{MOB3}	T_{MOB3}	T_{MOB1}	T_{MOB2}	T_{MOB3}
Φ_{Fe}		0.985	0.985	0.880	0.935	0.985	0.950	0.970	0.950	0.999	0.860	0.870
Φ_{Si}		0.085	0.085	0.030	0.015	0.170	0.088	0.050	0.080	0.200	0.041	0.038
H_2O (wt%)		1.60	1.00	0.88	0.88	1.07	0.95	0.90	0.92	0.88	0.76	0.78
Impactor core mass fraction		0.334	0.336	0.289	0.303	0.357	0.325	0.323	0.323	0.370	0.285	0.288
Impactor core radius (km)		276.6	277.1	263.5	306.6	407.6	274.1	273.5	183.2	412.6	262.4	263.2
Impactor silicate (wt%)	FeO	0.87	0.87	6.41	3.55	0.92	2.86	1.70	2.85	0.06	7.45	6.95
	SiO_2	50.34	50.81	49.41	51.29	48.33	49.74	51.37	49.98	47.98	48.68	49.01
	MgO	38.02	38.37	35.20	35.98	40.24	37.70	37.37	37.55	41.45	35.08	35.21
	Al_2O_3	4.63	4.67	4.29	4.38	4.90	4.59	4.55	4.57	5.05	4.27	4.29
	CaO	3.73	3.77	3.46	3.53	3.95	3.70	3.67	3.69	4.07	3.44	3.46
	H_2O	2.40	1.51	1.24	1.26	1.66	1.41	1.33	1.36	1.40	1.06	1.10
Impactor core (wt%)	Fe	88.16	88.16	89.98	91.19	84.40	87.69	89.69	88.07	83.33	89.21	89.46
	Ni	5.23	5.23	5.98	5.70	5.01	5.40	5.41	5.42	4.88	6.07	6.01
	Co	0.25	0.25	0.28	0.27	0.24	0.25	0.25	0.25	0.23	0.29	0.28
	O	0.00	0.00	0.00	0.00	0.00	0.00	0.00	0.00	0.00	0.00	0.00
	Si	4.36	4.36	1.76	0.84	8.35	4.66	2.65	4.25	9.57	2.44	2.24
	S	2.00	2.00	2.00	2.00	2.00	2.00	2.00	2.00	2.00	2.00	2.00
K_D(Ni)	a	0.098				0.46				1.06		
	b	3605				2700				1553		
	c	-57				-61				-98		
K_D(Co)	a	0.267				0.36				0.13		
	b	1744				1500				2057		
	c	-37				-33				-57		
K_D(O)	a	0.986				0.6				0.6†		
	b	-3275				-3800				-3800†		
	c	0				22				22†		
K_D(Si)	a	0.549				1.3				2.98		
	b	-12324				-13500				-15934		
	c	0				0				‡		

LogK_D is given by a + b/T + cP/T.

*with K_D given in Eq. 10, while other models are based on that in Eq. 2.

†ref. 3

‡LogK_D = a + b/T +($c_1P + c_2P^2 + c_3P^3$)/T; c_1 = -155(25), c_2 = 2.26(84) and c_3 = -0.011(7) (ref. 63)

Supplementary Table 4 Results of multi-stage core formation modeling

Model #	Core mass fraction	Earth silicate FeO wt%	Ni ppm	Co ppm	H_2O ppm	Earth core O wt%	Si wt%	S wt%	H wt%	Difference from target values*
S1	0.311	7.96	1989	102.9	687	2.89	6.53	2.15	0.56	0.0025
S1'†	0.318	7.54	1887	97.5	659	2.72	7.56	2.11	0.34	0.0086
S2	0.284	8.05	2018	109.7	702	1.64	1.91	2.03	0.33	0.0226
S3	0.288	7.84	2177	112.9	685	1.69	2.37	2.11	0.32	0.0380
F1	0.332	8.44	1627	87.8	707	2.02	11.40	2.15	0.34	0.0506
F2	0.303	7.73	2066	96.5	715	0.97	5.87	2.14	0.33	0.0124
F3-1	0.295	7.98	1947	95.0	698	0.91	4.53	2.19	0.32	0.0134
F3-2	0.300	7.94	2097	99.7	693	0.80	5.51	2.16	0.33	0.0119
R1	0.345	8.10	1852	161.8	678	1.95	13.15	2.14	0.27	0.3507
R2	0.281	7.90	1708	129.1	709	0.62	1.41	2.03	0.28	0.1063
R3	0.281	7.83	2050	143.5	715	0.63	1.44	2.05	0.29	0.1872
HOM-1‡	0.275	8.07	1579	129.0	-	1.19	1.01	2.00	0.00	0.1315
HOM-2‡	0.331	6.37	3581	202.0	-	2.24	9.91	2.00	0.00	1.6911
Present Earth	0.325	8.05	1960	105	>690					

Present-day Earth composition is from refs. 18 and 59.

*Defined as Σ((calculated value)/(present Earth value)-1)^2 for core mass fraction and mantle concentrations of FeO, Ni and Co.

†with K_D given in Eq. 10

‡Homogeneous accretion models by ref. 60

Supplementary Table 5 Chemical compositions (wt%) of stating material and silicate melt

	SM*	run #1	run #2	run #3	run #4	run #5	run #6
N†	28	8	8	8	8	8	8
SiO_2	49.64	36.14 (123)	38.54 (126)	32.67 (65)	37.59 (99)	34.26 (98)	40.17 (57)
TiO_2	1.64	1.73 (14)	1.89 (9)	3.08 (14)	1.73 (7)	1.96 (16)	2.49 (12)
Al_2O_3	14.88	14.78 (81)	13.96 (43)	19.34 (77)	18.71 (48)	18.63 (103)	11.32 (80)
FeO	11.43	29.43 (193)	26.10 (175)	24.47 (79)	22.89 (89)	21.01 (42)	23.27 (136)
MgO	8.51	9.33 (35)	9.31 (16)	11.03 (29)	11.34 (23)	11.82 (81)	8.91 (57)
CaO	10.55	4.15 (46)	4.60 (13)	4.53 (40)	4.06 (22)	5.02 (72)	8.40 (57)
Na_2O	2.90	3.24 (22)	2.82 (19)	3.68 (20)	2.35 (39)	3.66 (40)	3.80 (23)
K_2O	0.12	0.25 (1)	0.25 (4)	0.32 (3)	0.26 (4)	0.43 (6)	0.32 (3)
Total	99.67	99.05 (120)	97.48 (100)	99.11 (96)	99.28 (79)	96.80 (263)	98.67 (74)

Numbers in parentheses are one standard deviation in the last digits.

*~0.6 and ~1.0 wt% H_2O was added.

†Number of analyses

Supplementary Table 6 Chemical compositions (wt%) of quenched liquid metals

	run #1	run #2	run #3	run #4	run #5	run #6
N*	8	-	8	8	8	8
Fe	85.00 (83)	85.6 (11)	85.03 (54)	83.55 (84)	85.24 (188)	92.54 (55)
Si	2.27 (20)	3.2 (3)	2.16 (15)	4.73 (64)	1.52 (20)	0.52 (10)
O	12.14 (50)	11.2 (10)	11.33 (42)	8.70 (58)	8.92 (61)	4.48 (40)
Al	0.20 (2)	N.D. -	0.21 (3)	0.31 (1)	0.43 (21)	0.01 (1)
Mg	0.08 (3)	N.D. -	0.09 (1)	0.10 (1)	0.22 (18)	0.01 (1)
Total	99.70 (85)	100.00	98.82 (86)	97.39 (118)	96.34 (102)	97.56 (53)
C†	1.28 (2)	-	1.01 (7)	3.49 (10)	1.69 (13)	1.93 (2)

Obtained with EDS for run #2 and with EPMA for other runs

Numbers in parentheses are one standard deviation in the last digits.

*Number of analyses

†Measured separately without carbon-coating
