# Supplementary material for: Experimental evidence for hydrogen incorporation into Earth’s core
Source: Nat Commun. 2021 May 11;12:2588. doi: 10.1038/s41467-021-22035-0 (PMC8113257; doi:10.1038/s41467-021-22035-0)
Supplement: Supplementary file 2 — Description of Additional Supplementary Files [file 41467_2021_22035_MOESM2_ESM.pdf]

Description of Additional Supplementary Files

File name: Supplementary Data 1:
Description: Evolution of P, T, fO2 and the silicate (BSE) and core compositions in Model #S1
